# Supplementary material for: BR109, a Novel Fully Humanized T-Cell-Engaging Bispecific Antibody with GPRC5D Binding, Has Potent Antitumor Activities in Multiple Myeloma
Source: Cancers (Basel). 2023 Dec 9;15(24):5774. doi: 10.3390/cancers15245774 (PMC10741763; doi:10.3390/cancers15245774)
Supplement: Supplementary file 1 [file cancers-15-05774-s001.zip › cancers-2681963-supplementary.pdf]

# Supplementary Materials

**Table S1**. The purity of BR109 with various accelerated treatment conditions was verified by SEC.

| Sample | Multimer peak (%) | Main peak (%) | Fragment peak (%) |
|---|---|---|---|
| BR109 | 0.1 | 99.9 | 0 |
| BR109-HT | 0.1 | 96.9 | 2.9 |
| BR109-low pH | 0.5 | 99.3 | 0.2 |
| BR109-moderate pH | 0 | 96.9 | 3.1 |
| BR109-high pH | 0.3 | 95.7 | 4.0 |

**Table S2.** Expression levels of GPRC5D in different MM cell lines were detected by flow cytometry assay.

| Cell line | Concentration of BR109 (μg/mL) | | | |
|---|---|---|---|---|
| | 0 | 0.02 | 1 | 50 |
| MM.1R | 0.5%[1] | 98.7% | 100% | 97.9% |
| MM.1S | 0.3% | 68.6% | 98.4% | 98.5% |
| NCI-H929 | 0.3% | 42.8% | 82.2% | 97.4% |
| RPMI8226 | 0.4% | 19.0% | 64.7% | 96.9% |

[1] This value is the percentage of cells in the same fluorescence channel boundary (gate) to the total cells under the same experimental conditions.

**Table S3.** The efficacy results at the end of the dosing at day 18.

| Group | Antibody | Administration (mg/kg) | TV (mean±SEM[1]) $mm^3$ | T/C (%) | TGI (%) | pValue |
|---|---|---|---|---|---|---|
| 1 | PBS | 1.0 | 3303.18 ± 166.65 | - | - | - |
| 2 | BR109 | 1.0 | 85.62 ± 49.21 | 2.59 | 97.41 | <0.001 |
| 3 | BM03 | 1.0 | 2429.01 ± 264.07 | 73.54 | 26.46 | 0.087 |

[1] SEM: Standard Error of the Mean

**Table S4.** The tolerance of mice at the end of the dosing.

| Group | Antibody | Number of mice Day0/Day18 | Day 0 Body Weight (mean±SEM) g | Day 18 Body Weight (mean±SEM) g | Body Weight Change (mean±SEM) |
|---|---|---|---|---|---|
| 1 | PBS | 5/5 | 24.58 ± 0.65 | 25.78 ± 1.26 | -0.27% |
| 2 | BR109 | 5/5 | 25.37 ± 0.52 | 25.97 ± 0.77 | -1.71% |
| 3 | BM03 | 5/5 | 27.07 ± 0.6 | 30.05 ± 0.56 | -2.26% |

**Table S5.** The efficacy results at the end of the experiment at day 21.

| Group | Antibody | Administration (mg/kg) | TV (mean±SEM) $mm^3$ | T/C (%) | TGI (%) | pValue |
|---|---|---|---|---|---|---|
| 1 | IsotypeCD3 (i.v.) | 0.8 | 2416.58 ± 155.28 | - | - | - |
| 2 | BR109 (i.v.) | 0.05 | 59.45 ± 42.78 | 2.46 | 97.54 | <0.001 |

| | | | | | | |
|---|---|---|---|---|---|---|
| 3 | BR109 (i.v.) | 0.2 | 0 ± 0 | 0.00 | 100.00 | <0.001 |
| 4 | BR109 (i.v.) | 0.8 | 0 ± 0 | 0.00 | 100.00 | <0.001 |
| 5 | Talquetamab analog (i.v.) | 0.05 | 1240.41 ± 150.36 | 51.33 | 48.67 | 0.177 |
| 6 | Talquetamab analog (i.v.) | 0.2 | 99.29 ± 80.79 | 4.11 | 95.89 | <0.001 |
| 7 | BR109 (s.c.) | 0.2 | 0 ± 0 | 0.00 | 100.00 | <0.001 |

**Table S6.** The tolerance of mice at the end of the experiment.

| Group | Antibody | Number of mice Day0/Day18 | Day 0 Body Weight (mean±SEM) g | Day 21 Body Weight (mean±SEM) g | Body Weight Change (mean±SEM) |
|---|---|---|---|---|---|
| 1 | IsotypeCD3 (i.v.) | 5/5 | 24.8 ±0.50 | 27.8±0.54 | 12.34% |
| 2 | BR109 (i.v.) | 5/5 | 24.7 ±0.54 | 26.0±0.86 | 5.25% |
| 3 | BR109 (i.v.) | 5/5 | 24.0 ±0.51 | 24.1±0.57 | 0.58% |
| 4 | BR109 (i.v.) | 5/5 | 24.7 ±0.67 | 24.5±1.28 | -0.86% |
| 5 | Talquetamab analog (i.v.) | 5/5 | 24.3 ±0.79 | 24.4±1.47 | 1.18% |
| 6 | Talquetamab analog (i.v.) | 5/5 | 24.4 ±0.46 | 24.7±1.28 | 1.13% |
| 7 | BR109 (s.c.) | 5/5 | 24.5 ±0.55 | 25.7±0.80 | 4.90% |

**Table S7.** The binding activities of BR109 to HEK293T_hGPRC5D/cynoGPRC5D/mGPRC5D were determined by flow cytometry assay.

| $EC_{50}$ (nM) | HEK293T_hGPRC5D | HEK293T_cynoGPRC5D | HEK293T_mGPRC5D |
|---|---|---|---|
| BR109 | 1.97[1] | 38.15 | 389.8 |
| | 1.99 | 30.62 | 324.1 |

[1] The experiment was performed twice in duplicate.

| | BM02 -Alexa488 | 80A8 -Alexa488 | 155C2 -Alexa488 |
|---|---|---|---|
| BM02 | 83% | 57% | 35% |
| 80A8 | 100% | 99% | 29% |
| 155C2 | 89% | 73% | 94% |

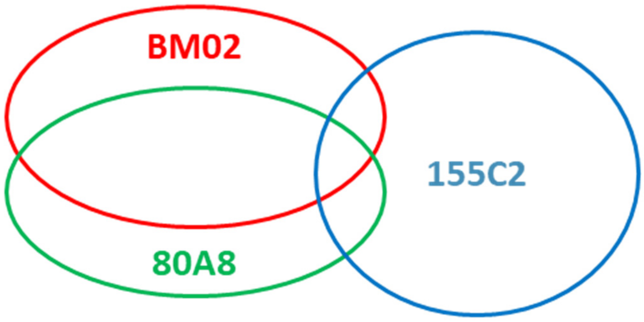


**Figure S1.** The epitope binning of anti-GPRC5D antibodies.

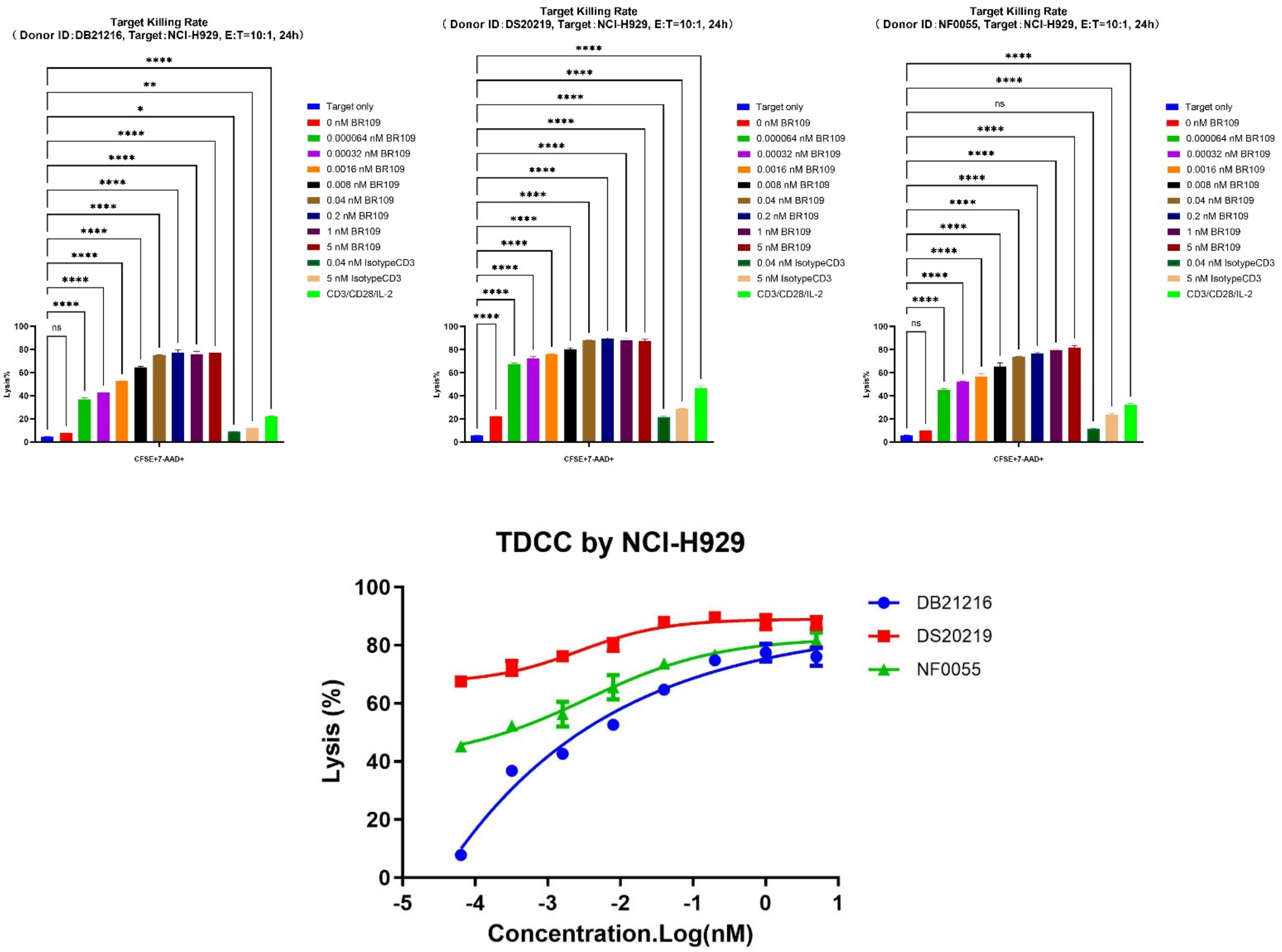


| | DB21216 | DS20219 | NF0055 |
|---|---|---|---|
| EC50 | ~ 8.281e-019 | 0.002676 | 0.003460 |

**Figure S2.** Cytotoxicity of GPRC5D×CD3 TCB BR109 when coculturing multiple myeloma cell line NCI-H929 with human PBMCs from three different donors.

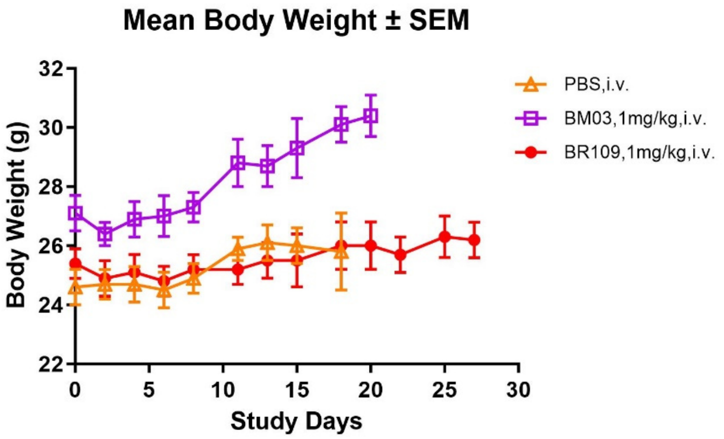


**Figure S3.** Body weights of NCI-H929 xenograft NPG mice with human immune cells (donor ID: DZ211314) treated with BR109 or BM03.

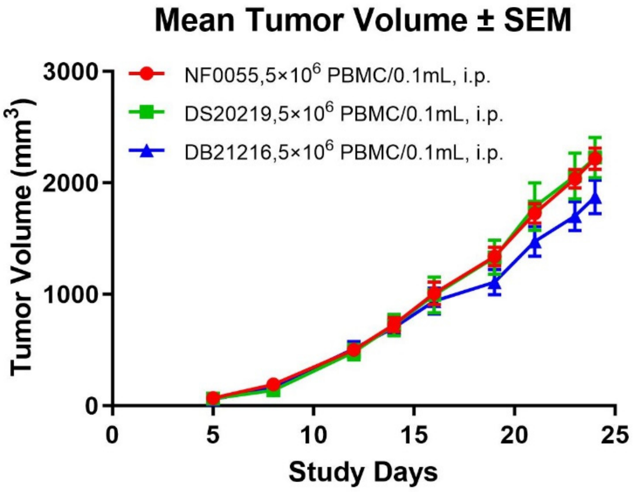


**Figure S4.** Tumor volumes of NCI-H929 xenograft NPG mice with human immune cells from three different donors (donor ID: NF0055, DS20219, DB21216). No drug was administered in this experiment.

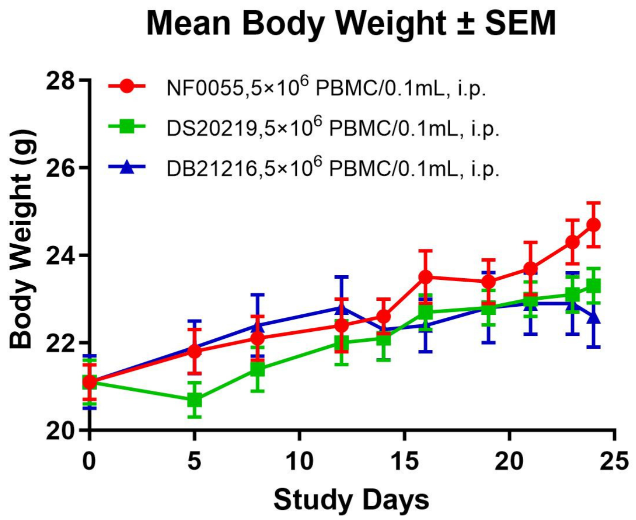


**Figure S5.** Body weights of NCI-H929 xenograft NPG mice with human immune cells from three different donors (donor ID: NF0055, DS20219, DB21216). No drug was administered in this experiment.

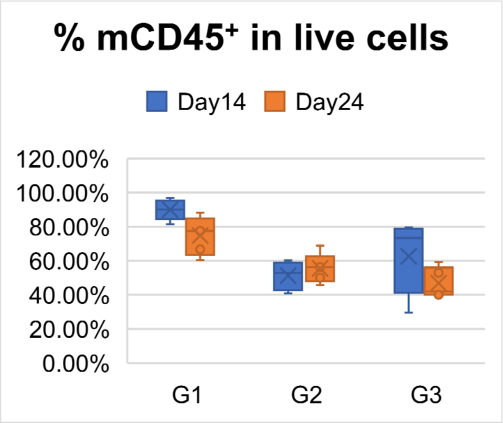


(a)

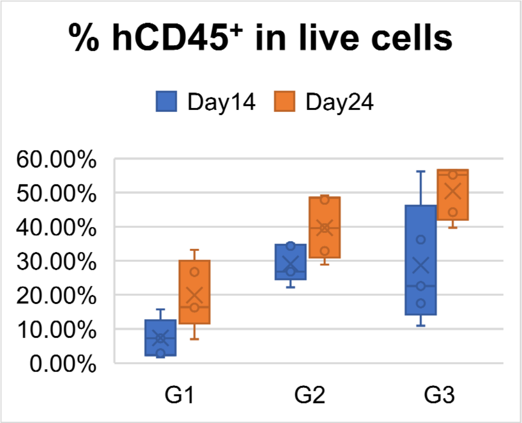


(b)

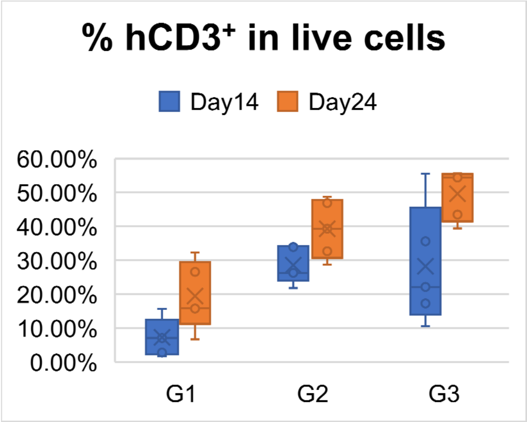


(c)

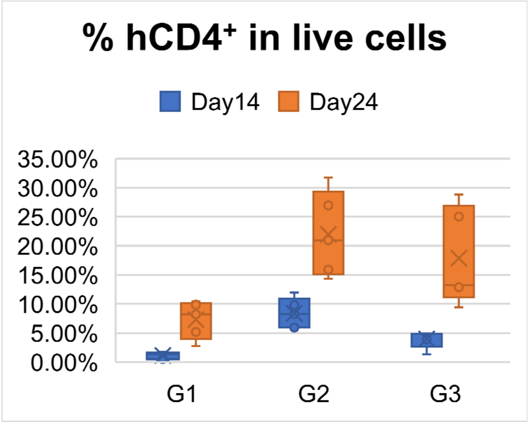


(d)

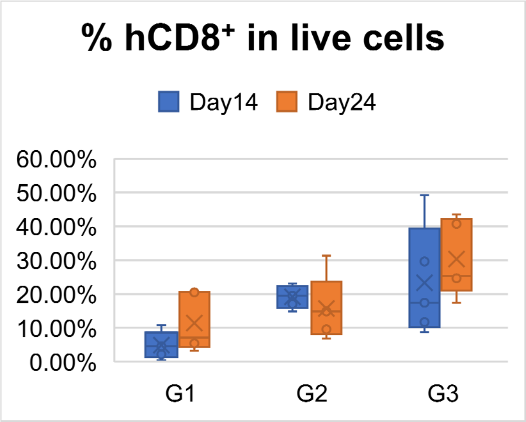


(e)

**Figure S6.** The reconstruction of human immune cells from three different donors, (a) mouse $CD45^+$ cell populations, (b) human $CD45^+$ cell populations, (c) human $CD3^+$ cell populations, (d) human $CD4^+$ cell populations, and (e) human $CD8^+$ cell populations in the NCI-H929 xenograft NPG mice models. G1: NF0055; G2: DS20219; G3: DB21216.

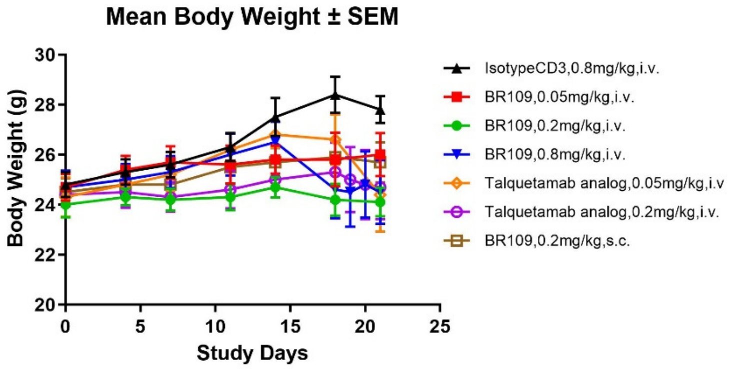


**Figure S7.** Body weights of NCI-H929 xenograft NPG mice with human immune cells (donor ID:DB21216) treated with BR109 or Talquetamab analog.
